# Acute Eosinophilic Pneumonia Associated With COVID-19 Infection

**DOI:** 10.7759/cureus.26501

**Published:** 2022-07-01

**Authors:** Derek C Vogel, Karim El-Kersh

**Affiliations:** 1 Pulmonary and Critical Care, University of Nebraska Medical Center, Omaha, USA

**Keywords:** pulmonary disease, systemic steroids, bronchoalveolar lavage, acute eosinophilic pneumonia, covid-19

## Abstract

The knowledge about COVID-19 infection and sequelae is evolving. Acute eosinophilic pneumonia (AEP) is not a well-recognized complication of COVID-19 infection, with few cases reported in the literature. We report a case of a 60-year-old male with a history of an orthotopic heart transplant on chronic immunosuppression who had AEP three weeks after a COVID-19 infection. He presented with diarrhea and acute kidney injury without respiratory symptoms. After discharge, the patient experienced progressive fevers, dyspnea, and cough resulting in a second admission to the hospital with acute hypoxic respiratory failure requiring supplemental oxygen. Imaging demonstrated ground-glass opacities with areas of consolidation and bronchoalveolar lavage fluid demonstrated AEP. The patient was treated with steroids resulting in the resolution of his symptoms and radiographic findings. This case highlights the potential for AEP to complicate COVID-19 infections.

## Introduction

Acute eosinophilic pneumonia (AEP) is an uncommon pulmonary disease. It presents with symptoms of acute fever, cough, and dyspnea with bilateral infiltrates on chest x-ray or chest computed tomography (CT). It is associated with inhalation exposures such as cigarette smoking, medications/toxins, and infections (commonly fungal or parasitic). The modified Philit criteria are used to diagnose “definite” AEP, which includes acute respiratory illness of less than or equal to one-month duration, pulmonary infiltrates on chest radiography or CT, pulmonary eosinophilia as demonstrated by more than 25% eosinophils in bronchoalveolar lavage (BAL) fluid or eosinophilic pneumonia on lung biopsy, and the absence of other specific pulmonary eosinophilic diseases [1]. AEP has been uncommonly reported in association with viral illnesses. We report a case of AEP in association with COVID-19 infection.

## Case presentation

A 60-year-old male was admitted to the hospital with acute fever, hypoxia, dyspnea, and cough three weeks after the diagnosis of COVID-19 in the fall of 2021. His medical history was significant for an orthotopic heart transplant in 2018 due to non-ischemic cardiomyopathy. He was taking 500 mg of mycophenolate mofetil twice a day, and 1.5 mg of tacrolimus in the morning and 1.0 mg in the evening. Twenty days prior, he was admitted with gastrointestinal complaints and acute kidney injury. He was found to be COVID-19 positive on screening with a reverse transcriptase-polymerase chain reaction (RT-PCR) nasal swab. He did not have respiratory symptoms or require oxygen and did not receive any COVID-19-specific therapy. He was discharged three days later in good condition.

**Figure 1:**
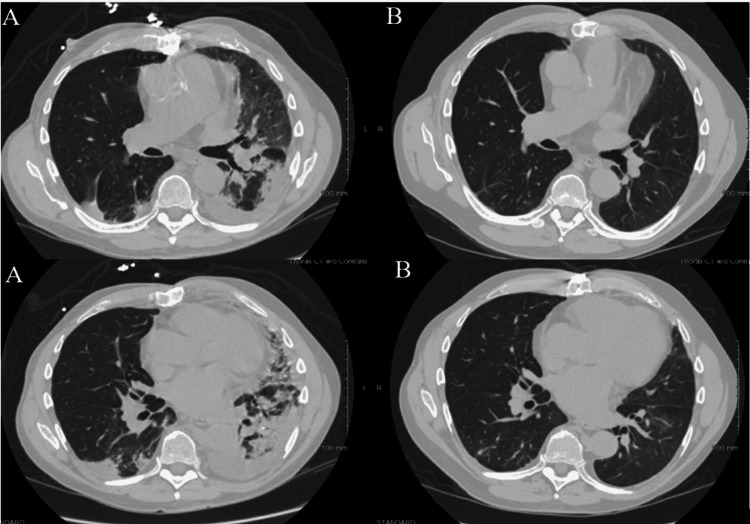
Chest CT upon presentation (A) shows left worse than right consolidative and ground-glass infiltrates and upon follow-up at six weeks (B) shows near-complete resolution of the infiltrates. CT: computed tomography.

On day three of his second admission, the pulmonary team was consulted. The patient was intermittently febrile, tachycardic, tachypneic, and on two liters of supplemental oxygen by a nasal cannula. His labs were notable for a white blood cell count of 14.7 x 10^3^/µL with 86% neutrophils and zero absolute eosinophils, procalcitonin 0.47 ng/ml, C-reactive protein (CRP) 20.2 mg/dL, and COVID-19 PCR positive at 36 cycle thresholds. Blood cultures and other infectious tests were unremarkable. Chest CT was notable for a left upper lobe ground-glass opacity (GGO), bibasilar dense consolidations, and a small left-sided pleural effusion (Figure 1A). He was started on intravenous fluids, vancomycin, cefepime, and azithromycin on admission. 

We performed a bronchoscopy with bronchoalveolar lavage (BAL) at the left lower lobe. About 25 ml of BAL fluid was obtained with 170 white blood cells and no epithelial cells. There were 37% eosinophils, 38% macrophages, 17% neutrophils, and 8% lymphocytes. We considered other etiologies of increased eosinophils, such as infections (parasitic/fungal) and asthma. Stool ova and parasite studies, BAL galactomannan, gram stain, acid-fast stain, and all cultures were negative. The serum immunoglobulin (Ig) E level was normal, and the patient lacked signs or symptoms of asthma. These findings were consistent with AEP and the patient was started on 60 mg of prednisone (approximately 1 mg/kg). He felt better the next day and was weaned off oxygen by day six of prednisone. After seven days of 60 mg of prednisone, his dose was decreased to 40 mg. Overall, he was instructed to take 40 mg for seven days, 30 mg for seven days, and then 20 mg until his six-week follow-up. At his six-week follow-up, he had improved significantly and had returned part-time to work. His CT scan at that time showed resolution of the consolidative process (Figure 1B), and he was slowly tapered off prednisone over the next three weeks.

## Discussion

This case highlights an unusual association of AEP with a COVID-19 infection and the diagnostic challenges of unusual pneumonia during the pandemic [2]. To our knowledge, this association between AEP and COVID-19 has been reported only in two other case reports (Table 1) [3,4].

**Table 1:** Demographic and clinical comparisons of three patients with acute eosinophilic pneumonia secondary to COVID-19. BAL: bronchoalveolar lavage.

	Age/sex	Medical history	Presentation	Diagnosis	Potential confounders	Treatment	Outcome
Case 1 [4]	77-year-old male	Asthma	Acute dyspnea and chest pain	BAL	Favipiravir and lascufloxacin	Prednisolone 0.5 mg/kg with prolonged taper	Significant improvement in imaging and symptoms at four weeks
Case 2 [3]	61-year-old male	None	Fever and dyspnea about four weeks after symptoms began	Biopsy	Hydroxychloroquine, azithromycin, and lopinavir/ritonavir	Methylprednisolone 60 mg per day followed by 30 mg prednisone per day on discharge	Significant improvement in imaging and symptoms at four weeks
Case 3 (Vogel)	60-year-old male	Heart transplant	Fevers and dyspnea three weeks after a positive test	BAL	Tacrolimus	One mg/kg prednisone with prolonged taper	Significant improvement in imaging and symptoms at six weeks

The first case was a 77-year-old male with a history of asthma who presented with chest pain and dyspnea. He was found to be COVID-19 positive and had bilateral pulmonary infiltrates on chest CT. He was treated with favipiravir and lascufloxacin. His peripheral eosinophils were 12.1% on admission and worsened during his hospital stay. Favipiravir or lascufloxacin could have caused a drug reaction, but the authors argued against this as he did not improve after they were discontinued. Eosinophils were seen in his BAL fluid, but a differential was not provided. The authors came to a diagnosis of AEP clinically. He was treated with steroids and improved [4].

The second case was a 61-year-old male without a medical history who presented with two weeks of fever, cough, and dyspnea. He was COVID-19 positive with bilateral pulmonary infiltrates. He was treated with hydroxychloroquine, azithromycin, and lopinavir/ritonavir. He was discharged but returned a week later with worsening symptoms. He was treated with methylprednisolone and ceftriaxone. He underwent BAL with transbronchial biopsies five days after admission. Only 5% of the white blood cells were eosinophils, but his biopsies showed eosinophils intermixed in the interstitial tissue and alveoli. Hydroxychloroquine, azithromycin, or lopinavir/ritonavir could have caused AEP, but the authors argued against this since he improved while still on them. They argued that the steroids likely contributed to the low eosinophilic count in his BAL fluid [3]. 

Our patient was not exposed to new medications or exposures prior to his presentation, making COVID-19 a more convincing etiology of AEP. Viral illnesses have infrequently been associated with AEP, but there are case reports of its association with influenza and human immunodeficiency virus (HIV) [5-7]. Some therapies for COVID-19 are novel or uncommon, and therefore it is difficult to conclude that they do not cause AEP. The website Pneumotox includes an association between tacrolimus and AEP, but this is rare [8]. Our patient had been on tacrolimus for over two years and improved on it, so the likelihood that this caused his episode is low.

Our patient’s presentation is like others with COVID-19. Weeks after his diagnosis, he presented with worsening respiratory symptoms and bilateral consolidations. These findings are suggestive of a superimposed bacterial infection or organizing pneumonia (OP). Given his lack of exposure and peripheral eosinophilia, we did not suspect AEP. If he hadn’t been immunocompromised with concerns for atypical infections, he could have been treated empirically with steroids for OP and clinically improved without us knowing the true cause. The incidence of AEP after COVID-19 infection could possibly be underestimated based on this case.

## Conclusions

Overall, the reported incidence of viral-associated AEP is low but growing. At this time, it is unclear why an acute viral illness would cause AEP, and more information is needed for clarification. Regardless, AEP should be on the differential diagnosis in patients with recent COVID-19 infections who present with worsening respiratory symptoms and pulmonary infiltrates. Early recognition and treatment with steroids can lead to a rapid and profound improvement in the patient’s condition.
